# In Silico and In Vitro Antiurolithic Effect of Extracts, Fractions, and Isolated Compounds From *Eugenia mattosii*

**DOI:** 10.1002/cbdv.202503734

**Published:** 2026-04-17

**Authors:** Bianca Letícia Maciél, Luma Da Silva Portella, Camile Cecconi Cechinel‐Zanchett, Anelize Dada, Rita de Cássia Vilhena da Silva, Anelise Felício Macarini, Valdir Cechinel Filho, Priscila de Souza, Giovana Vechi

**Affiliations:** ^1^ Nutrition Department Universidade do Vale do Itajaí UNIVALI Santa Catarina Brazil; ^2^ Pharmaceutical Sciences Universidade Do Vale do Itajaí UNIVALI Itajaí Brazil

**Keywords:** cryptostrobin, eugenia, oxalate, phytochemistry, pinostrobin

## Abstract

Urolithiasis, a condition characterized by the formation of calcium oxalate stones in the urinary tract, remains a significant clinical challenge due to the limited availability of effective preventive therapies. This study investigated the antiurolithic potential of different preparations from Eugenia mattosii, a plant rich in phenolic constituents. Calcium oxalate (CaOx) precipitation was induced in rat urine samples. Potassium citrate (10 mg/mL) was used as the positive control, while the crude methanolic extract (CME), chloroform fraction (CLF), ethyl acetate fraction (EAF), pinostrobin (PIN), and cryptostrobin (CRY) were tested at concentrations ranging from 0.01 to 1 mg/mL. CME and EAF increased CaOx crystal formation, whereas CLF, PIN, and CRY significantly reduced crystal formation at 0.01 mg/mL. In silico ADMET analysis supported favorable pharmacokinetic and safety profiles for PIN and CRY. Molecular docking revealed strong interactions with MMP‐9, suggesting a potential inhibitory effect that may reduce tissue injury and crystal nucleation. Overall, CLF and its flavonoids demonstrate promising antiurolithic activity.

## Graphical abstract

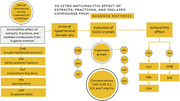

## 1 Introduction

Natural products play a significant role in the discovery and development of new drugs and effective therapeutic agents for the treatment of various diseases [1, 2]. Medicinal plants are widely used in the management of various pathologies, including urolithiasis [3].

Urolithiasis is characterized by the formation of crystals in the urinary tract, with calcium oxalate (CaOx) being the most common chemical composition. It is considered a growing public health problem affecting approximately 12% of the world's population and represents the third most common cause of urinary tract diseases, after infections and prostate disorders. Among all urinary system diseases, urinary stones are a major cause of morbidity [4]. There are still no effective strategies for the pharmacological dissolution of these crystals, and their management is primarily based on physical removal and preventive measures, especially in patients with recurrent disease. Currently, prevention focuses on dietary modification, increased fluid intake, and the appropriate use or avoidance of certain medications [5].

The genus Eugenia belongs to the Myrtaceae family and comprises more than 500 species, approximately 400 of which are found in Brazil [6]. This genus is home to species with high pharmacological potential, such as *E. fruticosa, E. uniflora, E. jambolone, E. brasilienses, E. dysenterica*, and *E. umbelliflora*. Some of the biological properties of these species are antibacterial, antiproliferative, gastroprotective, and antioxidant activity [7, 8, 9].

*Eugenia mattosii* is native to southern Brazil [10, 11]. To date, promising in vitro results have been reported with extracts and fractions, which showed significant antimycobacterial activity, especially in the chloroform fraction (CLF), an effect that was influenced by the presence of the isolated compounds pinostrobin and cryptostrobin [12]. In addition, ethyl acetate extracts and fractions showed promising results in anticholinesterase action related to the treatment of Alzheimer's [13]. Still, the crude extract and the evaluated fractions indicated promising antinociceptive activity, as did avicularin isolated from the ethyl acetate fraction (EAF) of the leaves. Additionally, the extracts, as well as the ethyl acetate and chloroform fractions (CLFs), and the respective substances isolated from these fractions, (−)‐catechin and cryptostrobin, demonstrated significant vasorelaxant effects in the presence and absence of functional endothelium, both in spontaneously hypertensive animals (SHR) and in normotensive animals [14], but nothing is known about their antiurolithic activity.

Considering the well‐documented phytochemical richness and pharmacological relevance of the genus Eugenia, the present study aimed to evaluate the antiurolithic potential of extracts, fractions, and the isolated compounds pinostrobin and cryptostrobin from Eugenia mattosii through an integrated approach. In addition to the in vitro assessment of calcium oxalate crystallization, in silico ADMET analyses were performed to predict the pharmacokinetic behavior and safety profiles, while molecular docking studies were conducted to explore putative molecular targets related to crystal formation and tissue injury.

## 2 Results and Discussion

From the initial column chromatography of the CLF, which resulted in the collection of 62 subfractions, similar subfractions were combined based on their thin‐layer chromatography (TLC) profiles, eluted with a hexane:acetone gradient, and developed with anisaldehyde reagent.

**FIGURE 1:**
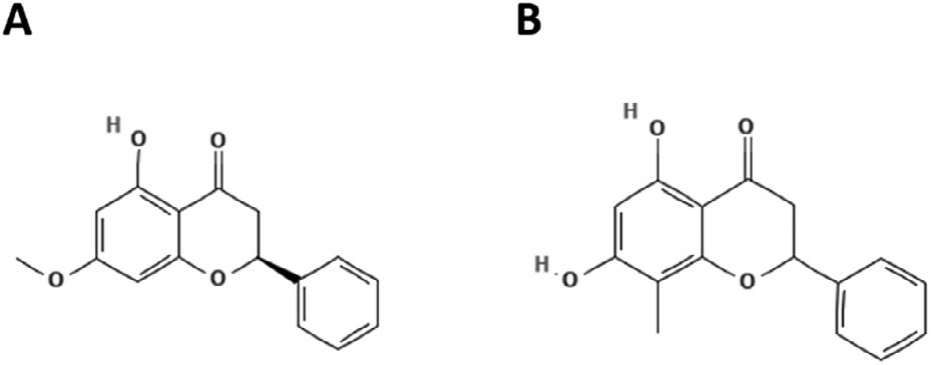
Chemical structure of compounds isolated from *E. mattosii* leaves. A) (2S)‐5‐hydroxy‐7‐methoxy‐2‐phenyl‐2,3‐dihydrochromen‐4‐one: pinostrobin; B) 5,7‐dihydroxy‐8‐methyl‐2‐phenyl‐2,3‐dihydrochromen‐4‐one: cryptostrobin.

Some subfractions (37‐57 and 106‐54) were rechromatographed following the same procedure. Subfractions 13‐30 derived from subfraction 37‐57 provided the isolation of 190 mg of a pure white solid, identified as (2S)‐5‐hydroxy‐7‐methoxy‐2‐phenyl‐2,3‐dihydrochromen‐4‐one—pinostrobin (Figure 1A). Subfractions 106‐154 were rechromatographed twice, providing the isolation of subfraction 3‐7 (78 mg), being another pure white solid, identified as 5,7‐dihydroxy‐8‐methyl‐2‐phenyl‐2,3‐dihydrochromen‐4‐one—cryptostrobin (Figure 1B). The identifications were carried out through TLC compared to the patterns previously isolated and identified by Nuclear Magnetic Resonance (NMR) [12].

**FIGURE 2:**
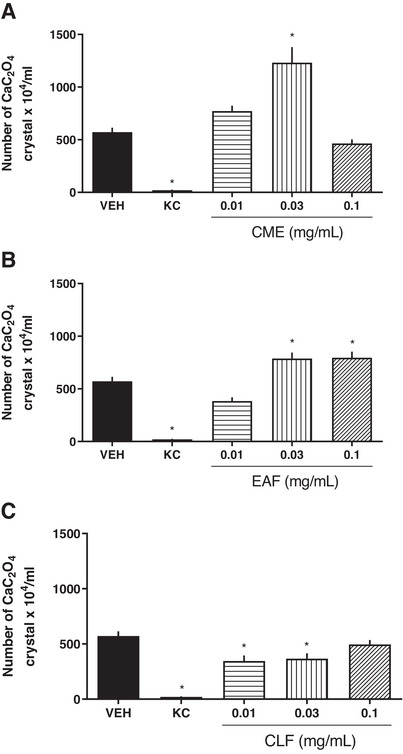
Effects of CME (A), EAF (B) and CLF (C) from *E. mattosii* leaves, at different concentrations, on CaOx crystals formation. VEH: vehicle, negative control; KC: positive control, potassium citrate 10 mg/mL; CME: crude methanolic extract; EAF: ethyl acetate fraction; CLF: chloroform fraction of *E. mattosii*. Results expressed as mean ± S.E.M. Statistical analyses were performed using one‐way ANOVA followed by Dunnett's test. *p < 0.05 versus VEH.

Regarding the EAF, 104 subfractions were collected in the first column and combined according to similarity. In this case, they were eluted with chloroform:methanol gradient and developed with ferric chloride (FeC). Subfraction 78‐91 allowed the isolation of quercetin (25.08 mg). Quercetin alone was not evaluated in this study since Park et al. [15] have already investigated the effects of quercetin on oxalate‐induced renal tubular cell injury and the inhibitory effects of quercetin on the formation of urinary crystal deposits in an animal model and concluded that quercetin has an inhibitory effect on the formation of urinary crystal deposits. Despite using different methodologies, our study demonstrated that the quercetin‐containing fraction was not effective in the in vitro analysis of antiurolithic activity (Figure 2B).

Figure 2 shows the results of the in vitro analysis of antiurolithic activity with the treatment of extract and fractions derived from the leaves of *E. mattosii*. In this figure, it is observed that both the Crude Methanolic Extract (CME) and the EAF promoted an increase in kidney stones at all concentrations tested. However, the CLF significantly reduced the number of CaOx crystals compared with the vehicle‐treated group. It is important to emphasize that potassium citrate (KC) was used as the positive control in this assay, given its well‐established role in the prevention of calcium stone formation [16].

**FIGURE 3:**
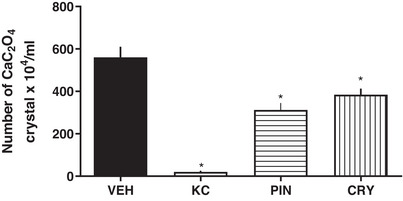
Effects of PIN and CRY compounds on CaOx crystals formation. VEH: vehicle, negative control; KC: positive control, potassium citrate 10 mg/mL; PIN: pinostrobin (0.01 mg/mL); CRY: cryptostrobin (0.01 mg/mL), compounds isolated from the CLF of *E. mattosii*. Results expressed as mean ± S.E.M. Statistical analyses were performed using one‐way ANOVA followed by Dunnett's test. *p < 0.05 versus VEH.

Thus, the compounds isolated from the CLF were evaluated to determine whether they were primarily responsible for the observed activity. Figure 3 shows the results obtained at the lowest effective concentration (0.01 mg/mL), at which both PIN and CRY significantly inhibited calcium oxalate crystal formation.

**FIGURE 4:**
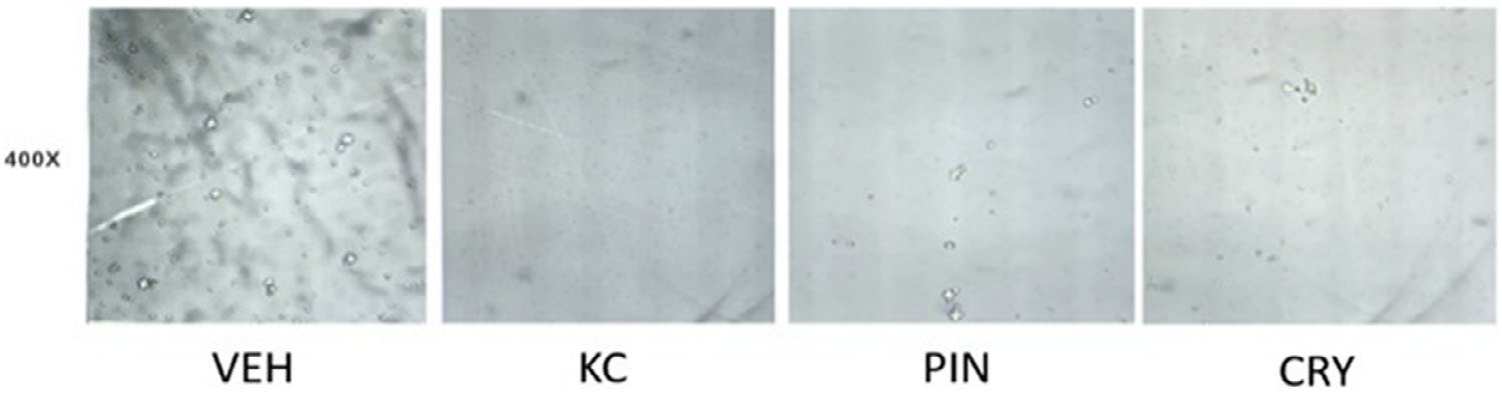
Representative images of CaOx crystals in the urine of hypertensive animals after in vitro treatment with PIN and CRY. VEH: vehicle, negative control; KC: positive control, potassium citrate 10 mg/mL; PIN: pinostrobin 0.01 mg/mL; CRY: cryptostrobin 0.01 mg/mL. Results were expressed as mean ± S.E.M. Statistical analyses were performed using one‐way ANOVA followed by Dunnett's test. *p < 0.05 versus vehicle.

These findings are further illustrated in Figure 4, which presents the microscopic images obtained after crystal precipitation. Taken together, the data demonstrate, for the first time, the ability of the CLF and its isolated compounds to markedly reduce CaOx crystallization in vitro, even at low concentrations.

It is worth noting that flavonoids are a large group of plant polyphenols with presumed beneficial effects on several common diseases, and recent studies have shown that plant flavonoids can effectively inhibit the formation of CaOx stones in vitro and in vivo, correlating with its diuretic, antioxidant, anti‐inflammatory, antibacterial, and other protective effects [17].

No other studies related to the genus *Eugenia* were found in the scientific literature regarding its antiurolithic action. However, some plants rich in phenolic compounds, the same class of compounds as pinostrobin and cryptostrobin, have already been described for their antiurolithic capacity. For instance, Bawari, Sah, and Tewari [18] investigated the antiurolithic potential of *Daucus carota* root extract against in vitro CaOx formation. The conclusion of the study was that *D. carota* has significant antiurolithiasis activity, which can be attributed to its content of saponins, tannins, flavonoids, and polyphenolics.

Mandal et al. [19] conducted a study to evaluate the in vivo antiurolithic activity of a fraction of ethyl acetate from *Aerva lanata* derived from the hydromethanolic extract of its aerial parts and demonstrated a significant antiurolithic effect through the restoration of the balance between urinary promoters and inhibitors, along with an improvement of urinary pH. This effect was related to the phenolic and flavonoid compounds present in the extract. Finally, Korah et al. [20] demonstrated diuretic and antiurolithiasis activity of the ethanolic extract of *Annona squamosa* leaves in experimental animals. Preliminary phytochemical analysis showed that the leaves contain several phytoconstituents, including carbohydrates, flavonoids, triterpenoids, tannins, phenolic compounds, and saponins; however, the diuretic properties have been primarily attributed to flavonoids and saponins.

The in silico analyses provided relevant preliminary information regarding the pharmacokinetic suitability and safety profile of PIN and CRY. High gastrointestinal (GI) absorption indicates favorable oral bioavailability potential. Blood–brain barrier (BBB) permeability predicts the ability of the compound to reach the central nervous system. P‐glycoprotein (P‐gp) substrate status is associated with efflux liability and potential reduction in intracellular exposure. In the BOILED‐Egg model, compounds located in the white region are predicted to have high passive GI absorption, whereas those in the yellow region are predicted to be BBB permeant. Collectively, these predictive criteria provide an in silico estimation of pharmacokinetic behavior.

As shown in Table 1, both compounds fully comply with Lipinski's rule of five, suggesting favorable oral bioavailability. Their molecular weights, hydrogen bond acceptor/donor counts, and lipophilicity values fall within the optimal range for passive diffusion. In addition, both molecules present high predicted gastrointestinal absorption, which further supports their potential for oral administration.

**TABLE 1:** Predictive criteria for assessing the oral bioavailability of pinostrobin and cryptostrobin.

	Lipinski					Volume (A^3^)	TPSA (A^2^)	GA	BBB	P‐gp	PPB
	MW	nHA	nHD	MLogP	Violation						
PIN	270.28	4	1	3.62	0	276.329	55.76	High	Yes	No	98.2
CRY	270.28	4	2	3.32	0	276.329	66.76	High	Yes	No	98.2

*Note*: Molecular Weight (MW; g/mol); Number of hydrogen bond acceptors (nHA); Number of hydrogen bond donors (nHD); logarithm of the partition coefficient(MLogP); Topological Polar Surface Area (TPSA); Gastrointestinal Absorption (GA); Blood‐ brain barrier penetration (BBB); P‐glycoprotein substrate (P‐gp); Plasma Protein Binding (PPB).

The topological polar surface area (TPSA) values of the two flavonoids are relatively low, which is consistent with their predicted ability to cross the blood–brain barrier (BBB). While BBB permeability may not be directly relevant to their antiurolithic application, this characteristic indicates that the compounds exhibit physicochemical features compatible with efficient membrane permeation. Moreover, neither pinostrobin nor cryptostrobin is predicted to behave as a P‐glycoprotein substrate, suggesting that efflux mechanisms are unlikely to compromise systemic availability. The high predicted plasma protein binding (PPB) values for both compounds (>98%) are expected for lipophilic natural products and may influence free circulating concentrations; however, this feature does not preclude biological activity.

**FIGURE 5:**
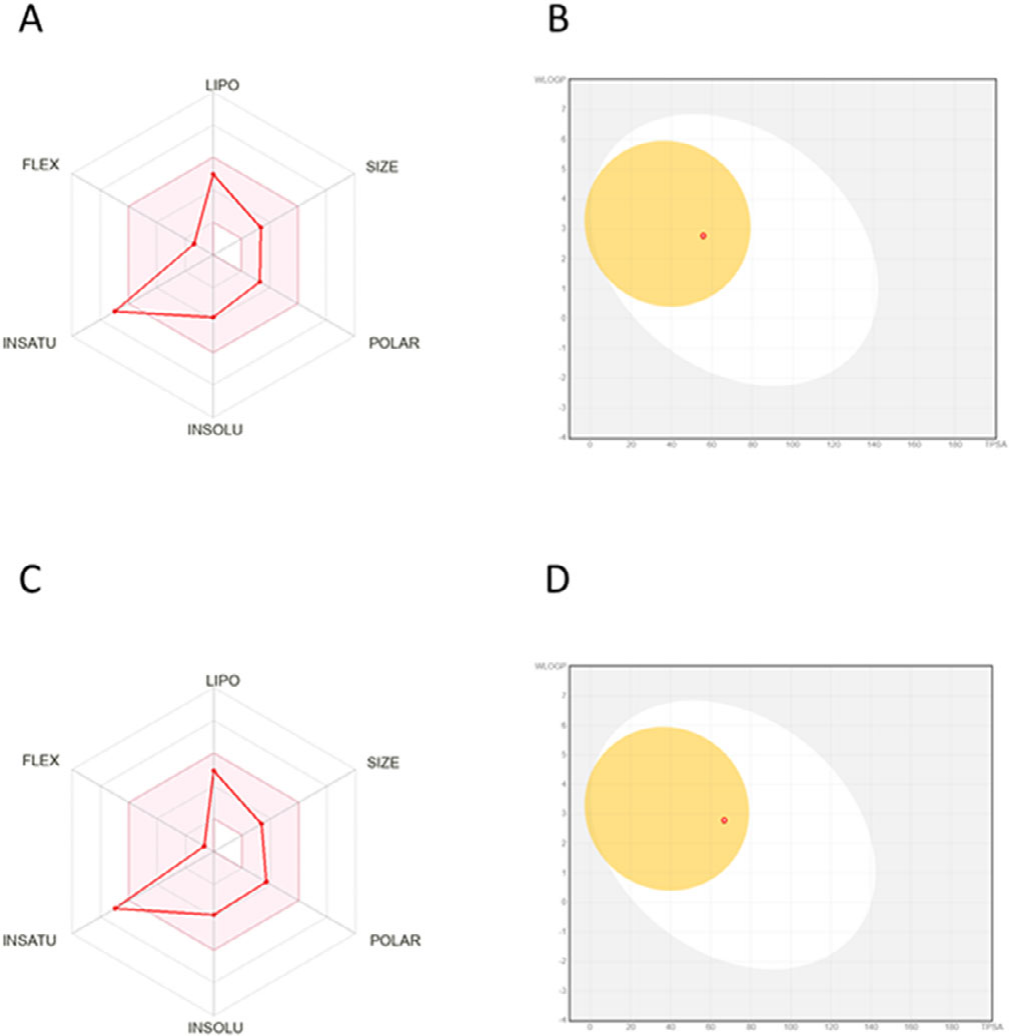
Radar plot and BOILED‐Egg analysis of the evaluated compounds: panels A and B correspond to pinostrobin, while panels C and D correspond to cryptostrobin, respectively. Radar plot and BOILED‐Egg diagram were generated using SwissADME web server.

Figure 5 illustrates the ADME predictions for the evaluated compounds, with panels A (radar plot) and B (BOILED‐Egg diagram) referring to pinostrobin and C (radar plot) and D (BOILED‐Egg diagram) correspond to cryptostrobin.

Regarding safety predictions (Table 2), both compounds demonstrated low to moderate probabilities for mutagenicity, carcinogenicity, and hepatotoxicity, with values within an acceptable range for early‐stage investigations. Differences between the two molecules were observed in specific endpoints. For instance, cryptostrobin showed a lower predicted probability of nephrotoxicity and neurotoxicity compared to pinostrobin, yet these variations remain theoretical and must be interpreted with caution. Importantly, none of the predicted risks reached values that would contraindicate the continuation of experimental evaluation.

**TABLE 2:** Predictive criteria for assessing the toxicological profile of pinostrobin and cryptostrobin.

	Mutagenicity (AMES)	Carcinogenicity	Hepatotoxicity	Nephrotoxicity	Neurotoxicity	Hematotoxicity	Respiratory
PIN	0.675	0.353	0.818	0.833	0.891	0.313	0.869
CRY	0.673	0.548	0.735	0.484	0.271	0.300	0.800

Regarding safety predictions (Table 2), both compounds exhibited low to moderate predicted probabilities for the evaluated toxicological endpoints. In general, probability values below 0.70 are considered indicative of moderate to low risk, whereas values between 0.70 and 0.90 suggest moderate to high concern that warrants further investigation. PIN showed higher predicted probabilities for hepatotoxicity (0.818), nephrotoxicity (0.833), neurotoxicity (0.891), and respiratory toxicity (0.869), although these values remain within the acceptable range for early‐stage screening. CRY presented comparatively lower probabilities for nephrotoxicity (0.484) and neurotoxicity (0.271), suggesting a more favorable theoretical safety profile in these specific endpoints. Importantly, neither compound reached probability values close to 1.0 that would indicate a high toxicological risk. Therefore, the in silico predictions support the continued experimental evaluation of both molecules while highlighting endpoints that merit closer monitoring in future in vitro and in vivo studies.

Taken together, these in silico results support the pharmacokinetic feasibility of PIN and CRY and suggest that both compounds have acceptable preliminary safety profiles. While these predictions cannot replace empirical testing, they provide a useful foundation for guiding subsequent in vitro and in vivo studies aimed at validating their antiurolithic potential.

The predicted binding affinities of pinostrobin and cryptostrobin for each selected protein target are summarized in Table 3. The selection of the molecular targets was guided by their reported direct or indirect involvement in calcium oxalate (CaOx) nucleation and urolithiasis‐related pathways. In this context, matrix metalloproteinases MMP‐2 (PDB: 8H78) and MMP‐9 (PDB: 4XCT) were included due to their roles in extracellular matrix remodeling and crystal adhesion processes. Glycolate oxidase (GO; PDB: 2RDT) was selected based on its key participation in oxalate biosynthesis, while phosphoethanolamine cytidylyltransferase (PDB: 3ELB) was considered for its metabolic relevance. Additionally, the calcium‐sensing receptor (CaSR) was evaluated in both its Venus flytrap (VFT) domain (PDB: 5FBK) and 7‐transmembrane (7TM) domain (PDB: 7DD7), given its central role in calcium homeostasis and signaling pathways associated with stone formation. Collectively, this panel of targets provides a mechanistically coherent framework to explore potential multi‐target effects of the investigated flavonoids in the context of CaOx crystallization.

**TABLE 3:** Binding affinity values (kcal/mol) of pinostrobin and cryptostrobin toward selected enzymes and receptors obtained by molecular docking, with redocking validation (RMSD) of the co‐crystallized ligand.

		Binding affinity (kcal/mol)			
Enzyme	PDB ID	Pinostrobin	Cryptostrobin	Co‐crystalized ligand	RMSD (Å)
MMP‐2	8H78	−9.1	−9.0	−9.4	0.978
MMP‐9	4XCT	−10.4	−10.2	−9.8	0.654
Glycolate Oxidase	2RDT	−7.8	−6.4	−7.3	0.269
Phosphoethanolamine Cytidylyltransferase	3ELB	−8.5	−8.4	−9,6	0.446
CaSR VFT domain	5FKB	−9.3	−9.3	−8.5	0.581
CaSR 7TM domain	7DD7	−7.8	−8.6	−9.5	1.695

The validation of the molecular docking protocol was performed through redocking of the crystallographic ligands in the respective target proteins. The superposition between the crystallographic and redocked poses, as well as the corresponding RMSD and binding affinity values, is presented in the Supplementary Material (Figures S1–S6).

Among all interactions, the most favorable binding affinity was observed between pinostrobin and the catalytic domain of MMP‐9 (PDB ID: 4XCT). Nonetheless, cryptostrobin exhibited nearly equivalent affinities across most macromolecules, consistent with its close structural similarity to pinostrobin.

**FIGURE 6:**
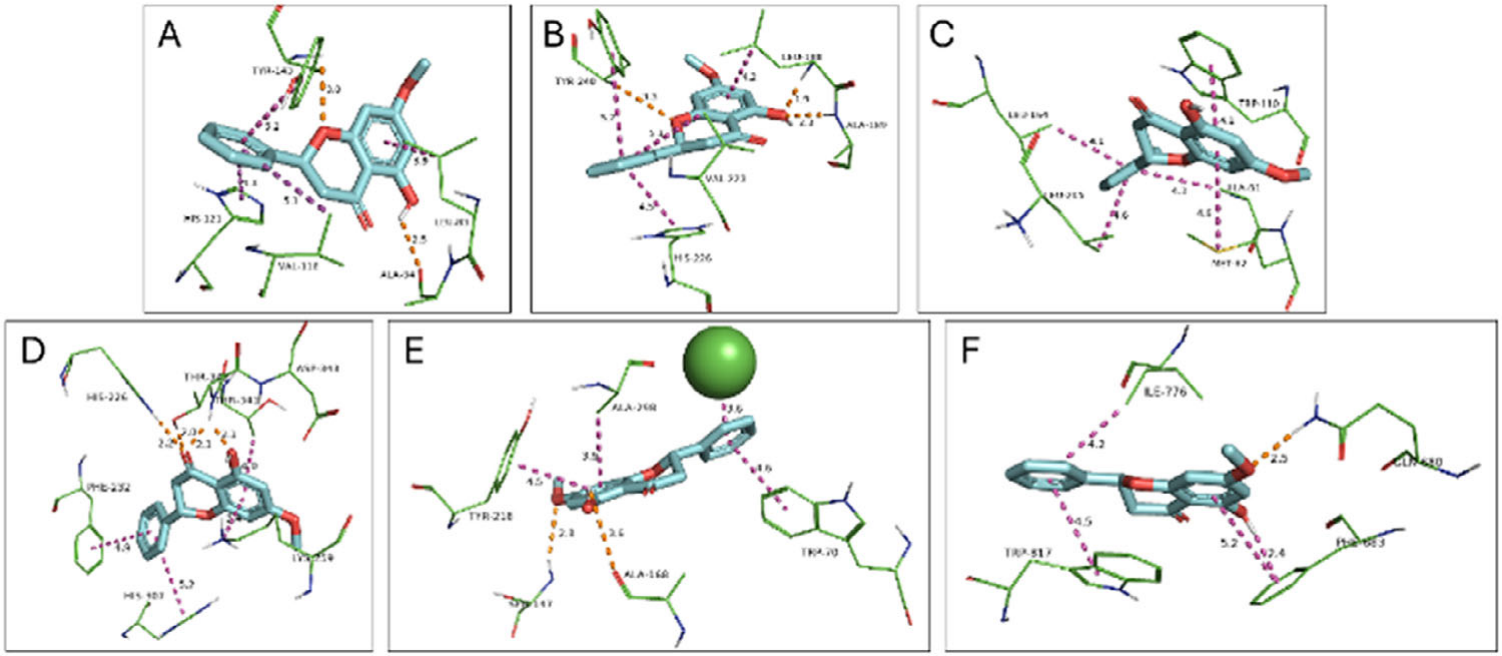
Molecular docking interactions of pinostrobin with target proteins involved in extracellular matrix remodeling, oxalate metabolism, phospholipid biosynthesis, and calcium signaling. Panels (A–F) depict the predicted binding poses and key amino acid interactions of pinostrobin within the active or functional domains of the following proteins: (A) Matrix metalloproteinase‐2 (PDB ID: 8H78; binding affinity: −9.1 kcal/mol), (B) Matrix metalloproteinase‐9 (PDB ID: 4XCT; −10.4 kcal/mol), (C) Glycolate oxidase (PDB ID: 2RDT; −7.8 kcal/mol), (D) Phosphoethanolamine cytidylyltransferase (PDB ID: 3ELB; −8.5 kcal/mol), (E) Calcium‐sensing receptor VFT domain (PDB ID: 5FKB; −9.3 kcal/mol), and (F) Calcium‐sensing receptor 7TM domain (PDB ID: 7DD7; −7.8 kcal/mol). Hydrogen bonds are represented in orange, while hydrophobic interactions are shown in magenta.

**FIGURE 7:**
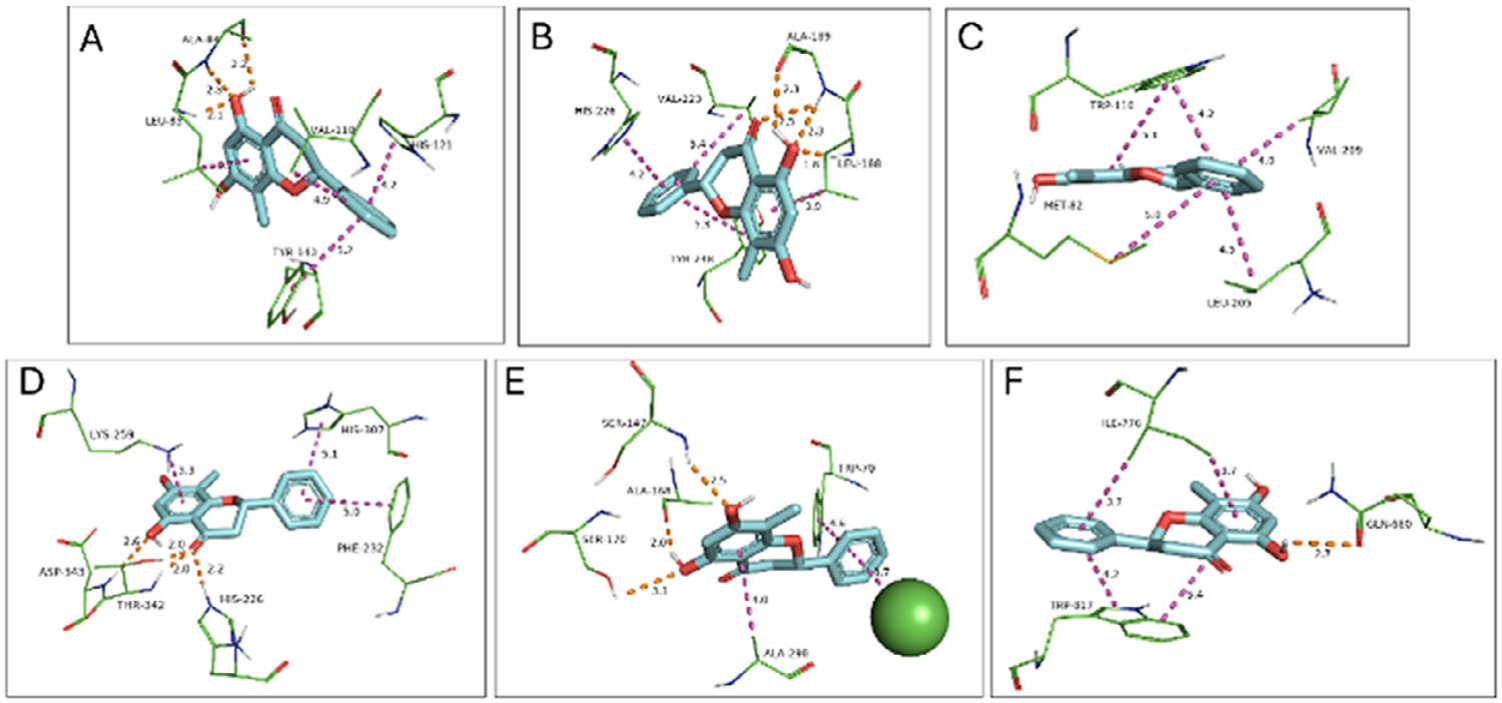
Molecular docking interactions of cryptostrobin with selected protein targets. Panels (A–F) illustrate the predicted binding conformations and principal residue interactions of cryptostrobin with: (A) Matrix metalloproteinase‐2 (PDB ID: 8H78; −9.0 kcal/mol), (B) Matrix metalloproteinase‐9 (PDB ID: 4XCT; −10.2 kcal/mol), (C) Glycolate oxidase (PDB ID: 2RDT; −6.4 kcal/mol), (D) Phosphoethanolamine cytidylyltransferase (PDB ID: 3ELB; −8.4 kcal/mol), (E) Calcium‐sensing receptor VFT domain (PDB ID: 5FKB; −9.3 kcal/mol), and (F) Calcium‐sensing receptor 7TM domain (PDB ID: 7DD7; −8.6 kcal/mol). Hydrogen bonds are depicted in orange and hydrophobic contacts in magenta.

The key MMP‐9 residues contributing to the stabilization of the ligand‐protein complex were identified as Leu222, Val223, Ala242, Met247, and Tyr248, which collectively promote a strong interaction [28]. The reference ligand of the selected PDB structure (4XCT), the hydroxamate‐based inhibitor ARP101, forms hydrogen bonds with Leu188 and Ala189 and coordinates the catalytic Zn^2^^+^ ion, in addition to establishing hydrophobic contacts with Ala189, His226, His230, Leu187, Val223, and Met247. In a comparable fashion, both pinostrobin and cryptostrobin interact with MMP‐9 through similar residues. Specifically, PIN presents H‐bonds with Tyr248, Ala189, and Leu188, and hydrophobic interactions with Met247, Val223, and His226, as shown in Figure 6. CRY presents H‐bonds with Ala189 and Leu188 and hydrophobic interactions with Val223, His226, and Tyr245, as shown in Figure 7.

Matrix metalloproteinases (MMPs), particularly MMP‐9, are zinc‐dependent endopeptidases implicated in extracellular matrix (ECM) remodeling, inflammation, and renal injury—processes that are central to the development of urolithiasis [29]. The strong binding affinities of pinostrobin and cryptostrobin toward MMP‐9, along with their capacity to form hydrogen bonds with key residues (Leu188 and Ala189), suggest potential inhibitory activity. However, their phenolic groups may exhibit limited Zn^2^^+^‐chelating capability, an important feature for effective MMP inhibition [28].

Previous research has shown that MMP‐9 expression is markedly increased in pathological conditions such as acute kidney injury, interstitial fibrosis, glomerulonephritis, and diabetic nephropathy [9]. Therefore, inhibition of MMP‐9 by pinostrobin and cryptostrobin could potentially mitigate tissue damage and reduce crystal nucleation, supporting their proposed antiurolithiatic effects.

In view of the lack of effective drug options for the treatment and prevention of urolithiasis, it is of paramount importance to search for more effective and safer therapeutic approaches, especially those based on natural products. This emphasizes the relevance of the present study and its fundamental role in the search for promising alternatives for its treatment and prevention. Although there are no previous studies on the specific antiurolithic action of *E. mattossi*, the results of this study are in line with research that highlights the potential of phenolic compounds, such as flavonoids, in preventing stone formation.

## 3 Conclusions

Based on the in vitro findings of this study, together with the in silico pharmacokinetic and toxicological predictions and the molecular docking results, the CLF of *Eugenia mattosii* and its isolated flavonoids, cryptostrobin and pinostrobin, emerge as promising natural candidates for preventing CaOx crystal formation. The favorable ADME and safety profiles, combined with their predicted ability to interact with MMP‐9, an enzyme implicated in renal tissue remodeling, reinforce their potential antiurolithiatic relevance. These results strengthen the prospect of identifying safer and more effective therapeutic options for this common and clinically challenging condition. Nevertheless, additional studies are required to elucidate the precise mechanisms of action, confirm their effects in vivo, and assess their potential clinical applicability.

## 4 Experimental Section

### 4.1 Phytochemical Analysis‐Isolation and Identification of Compounds

For this study, the crude extract and the chloroform and EAFs of the dried leaves of *Eugenia mattosii* previously prepared and described according to Vechi [12] were used. In order to isolate the main compounds, both fractions were subjected to chromatographic (CC) columns in glass columns with Macherey‐Nagel silica gel 60 (0.04–0.063 mm (bar) 230–400 mesh ASTM). 901 mg of the CLF was submitted to open CC in a 200 mL glass column with 39.525 g of silica gel 60 eluted with a hexane:acetone gradient, resulting in the collection of 62 subfractions in this initial column.

The similar subfractions of the first column were combined based on their TLC profiles, using pre‐coated Merck Kieselgel 60 F4 (0.25 mm) plates eluted with hexane:acetone gradient and developed with anisaldehyde reagent. Some subfractions were rechromatographed following the same procedure.

Ethyl acetate (EAF) fraction was submitted to chromatographic procedures in a 200 mL glass column with 65.45 g of silica gel 60 using a chloroform:methanol gradient.

### 4.2 Antiurolithic Activity

To obtain the urine, 6 female hypertensive rats (SHR), of the Wistar lineage, with 3 to 4 months of age, provided by the Univali Vivarium, were used. These animals were kept at a controlled room temperature (22°C ± 2°C), 12‐h light/dark cycle with free access to water and feed. All proposed methodologies and procedures were submitted for approval by the Ethics Committee on Animal Experimentation of Univali and were conducted under all established ethical standards (CEUA/UNIVALI: n° 050/18).

Before the experiment, they were weighed and left to fast for 8 h, with water at will. The animals received a gavage saline overload of 5 mL/100 g to obtain uniformity in sodium and water levels. The animals were then placed individually in metabolic cages, and urine was collected until completing 8 h.

Calcium oxalate (CaOx) precipitation was induced with the addition of 20 µL of 0.1 M sodium oxalate in 500 µL of urine at 37°C. For the urine assay, CaOx precipitations were induced in the negative control group (vehicle; VEH), positive control group (potassium citrate; KC; 10 mg/mL) and in the presence of extract and fractions from *E. mattosii* leaves: CLF, EAF, CME in different concentrations (0.01, 0.03, and 0.1 mg/mL), and isolated compounds of CLF pinostrobin (PIN), cryptostrobin (CRY) at the concentration of 0.01 mg/mL. After 60 min, the number of crystals at each concentration was evaluated in four fields, two in each sample, randomly chosen using a Neubauer chamber with a magnification of 400 x. The protocol as well as the readings were made in duplicate. The results were expressed as the mean ± the standard error of the mean (n = 5 – 8). Statistical analysis between groups was verified by one‐way analysis of variance (ANOVA). The analyses were performed using the GraphPad Prism software, version 9.5.1 for Windows.

### 4.3 In Silico Pharmacokinetic and Toxicological Evaluations

The pharmacokinetic characteristics of pinostrobin and cryptostrobin were assessed using the SwissADME online platform (available at https://www.swissadme.ch), which provides computational predictions for absorption, distribution, metabolism, and excretion parameters.

Toxicological properties were predicted with the ADMETlab 3.0 tool (accessible at https://admetlab3.scbdd.com), which offers in silico evaluation of a wide range of safety‐related endpoints.

The SMILES sequences used for these analyses were retrieved from the PubChem database (https://pubchem.ncbi.nlm.nih.gov).

### 4.4 Molecular Docking

Molecular docking for pinostrobin and cryptostrobin was conducted using crystal structures of selected enzymes, which were obtained from the Protein Data Bank (PDB) [21]. The proteins were prepared with AutoDock Tools (ADT 1.5.7) [22], where co‐crystal ligands, water, and unnecessary ions were removed, polar hydrogens were added, and Gasteiger charges were calculated. The 3D structure of pinostrobin and cryptostrobin were retrieved in SDF format from PubChem, and were prepared for docking using ADT 1.5.7. Docking was executed with AutoDock Vina [23], using a grid box centered on the active site with dimensions sufficient to accommodate ligand flexibility. Validation of the docking protocol was performed by redocking the native ligand and calculating RMSD (<2Å considered acceptable).

The potential interactions and 3D images were generated using PyMOL 3.0.3 (Schrodinger LLC, 2015), and 2D images were produced with the BIOVIA Discovery Studio program.

The selection of enzymes was predicated on their potential involvement in the CaOx nucleation process, directly or indirectly. The enzymes include the catalytic domain of metalloprotease (MMP)‐2 (PDB 8H78) and MMP‐9 (PDB 4XCT), Glycolate Oxidase (GO) (PDB 2RDT) [24], Phosphoethanolamine Cytidylyltransferase (PDB 3ELB) [25, 26], Calcium‐sensing receptor (CaSR) in the Venus flytrap (VFT) domain (PDB 5FBK), and the CaSR in the 7‐transmembrane (7TM) domain (PDB 7DD7) [27].

## Author Contributions

B. Letícia Maciél, L. Portela and G. Vechi prepared the extracts and performed the isolation of the compounds. A. Dada, C. Cecconi Cechinel‐Zanchett, P. de Souza and R. C. V. da Silva performed the experiments and analyzed the data. A.F. Macarini and P. de Souza performed the in silico analysis and molecular docking. P. de Souza and V. Cechinel Filho supervised and were responsible for the final version of the manuscript.

## Conflicts of Interest

The authors declare no conflicts of interest.

## Submission Policy

All data utilized in this study were generated internally, with no involvement of paper mills or other external entities that could manipulate research materials.

## Supporting information




**Supporting File 1**: cbdv71172‐sup‐0001‐SuppMat.docx

## Data Availability

The data supporting this study are available upon request to the corresponding author.
