# Supplementary material for: In Silico and In Vitro Antiurolithic Effect of Extracts, Fractions, and Isolated Compounds From Eugenia mattosii
Source: Chem Biodivers. 2026 Apr 17;23:e03734. doi: 10.1002/cbdv.202503734 (PMC13090003; doi:10.1002/cbdv.202503734)

**Supplementary material**

***In silico and In vitro* Antiurolithic Effect of Extracts, Fractions, and Isolated Compounds from *Eugenia mattosii***

Bianca Letícia Maciél ^a^, Luma Da Silva Portella ^a^, Camile Cecconi Cechinel-Zanchett ^a^, Anelize Dada ^b^, Rita de Cássia Vilhena da Silva ^b^, Anelise Felício Macarini ^b^, Valdir Cechinel Filho^b^, Priscila de Souza ^b*^, and Giovana Vechi ^a^

^a^ Nutrition department - Universidade do Vale do Itajaí UNIVALI (biancaleticia.maciel@gmail.com; luma.portella@outlook.com.br; camile@univali.br; giovanavechi@univali.br)

^b^ Pharmaceutical Sciences - Universidade do Vale do Itajaí UNIVALI (anelizedada.pos@gmail.com; ritasilva@univali.br; anelise.fm@univali.br; cechinel@univali.br; prisciladesouza@univali.br)

Correspondence:

Priscila de Souza

E-mail address: prisciladesouza@univali.br

Rua Uruguai, 458, Centro, 88302-901, Itajaí, Brazil.

Phone: +55 47 3341-7932, Fax: +55 47 3341-7744

https://orcid.org/0000-0002-5251-5642

Figure S1. **Redocking validation of the crystallographic ligand in MMP-2.**
Representation of the binding site of MMP-2 showing the superposition between the crystallographic ligand and the docked pose of (2~{R})-2-[[4-[(4-aminocarbonylphenyl)carbonylamino]phenyl]sulfonylamino]-5-[(2~{S},4~{S})-4-azanyl-2-[[(2~{S})-1-[[(2~{S})-1-[(5-azanyl-5-oxidanylidene-pentyl)amino]-5-oxidanyl-1,5-bis(oxidanylidene)pentan-2-yl]-methyl-amino]-4-methyl-1-oxidanylidene-pentan-2-yl]carbamoyl]pyrrolidin-1-yl]-5-oxidanylidene-pentanoic acid (L2U). The protein structure and the native co-crystallized ligand are shown in green, while the redocked ligand used for validation is displayed in blue. The redocking procedure yielded a binding affinity of −9.4 kcal/mol and a root mean square deviation (RMSD) of 0.978Å.
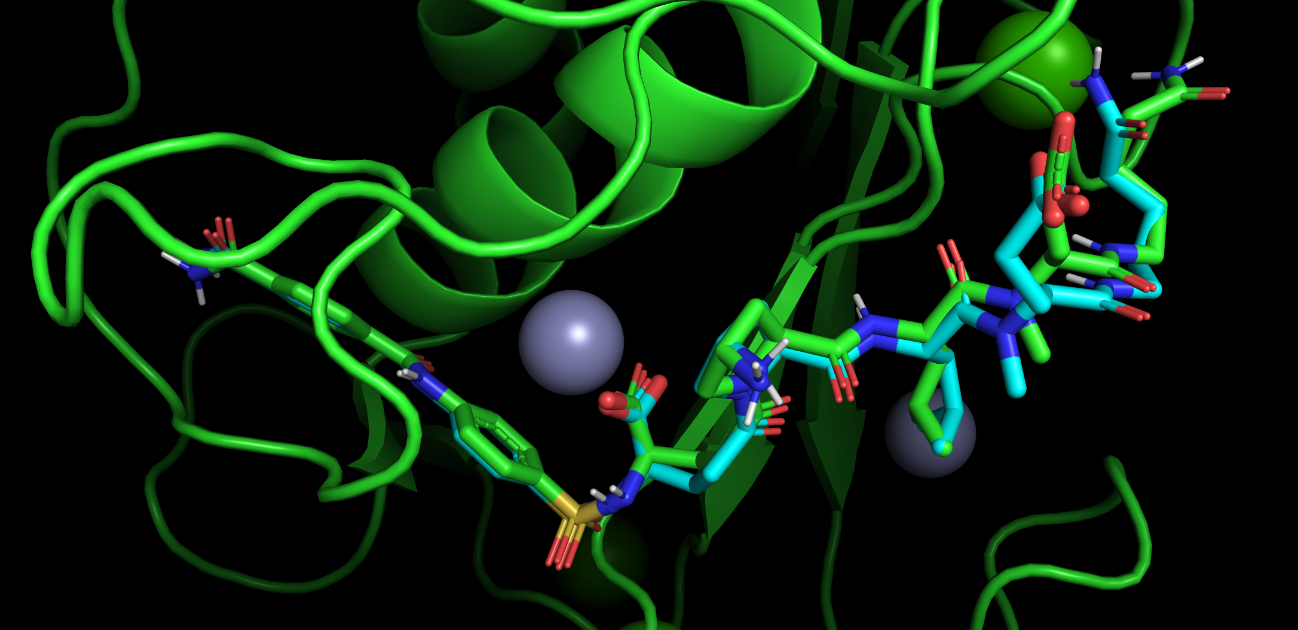


Figure S2. **Redocking validation of the crystallographic ligand in MMP-9.**
Representation of the binding site of MMP-9 showing the superposition between the crystallographic ligand and the docked pose of (2~{R})-3-methyl-~{N}-oxidanylidene-2-[(4-phenylphenyl)sulfonyl-propan-2-yloxy-amino]butanamide (N73). The protein structure and the native co-crystallized ligand are shown in green, while the redocked ligand used for validation is displayed in blue. The redocking procedure yielded a binding affinity of −9.8 kcal/mol and a root mean square deviation (RMSD) of 0.654Å.
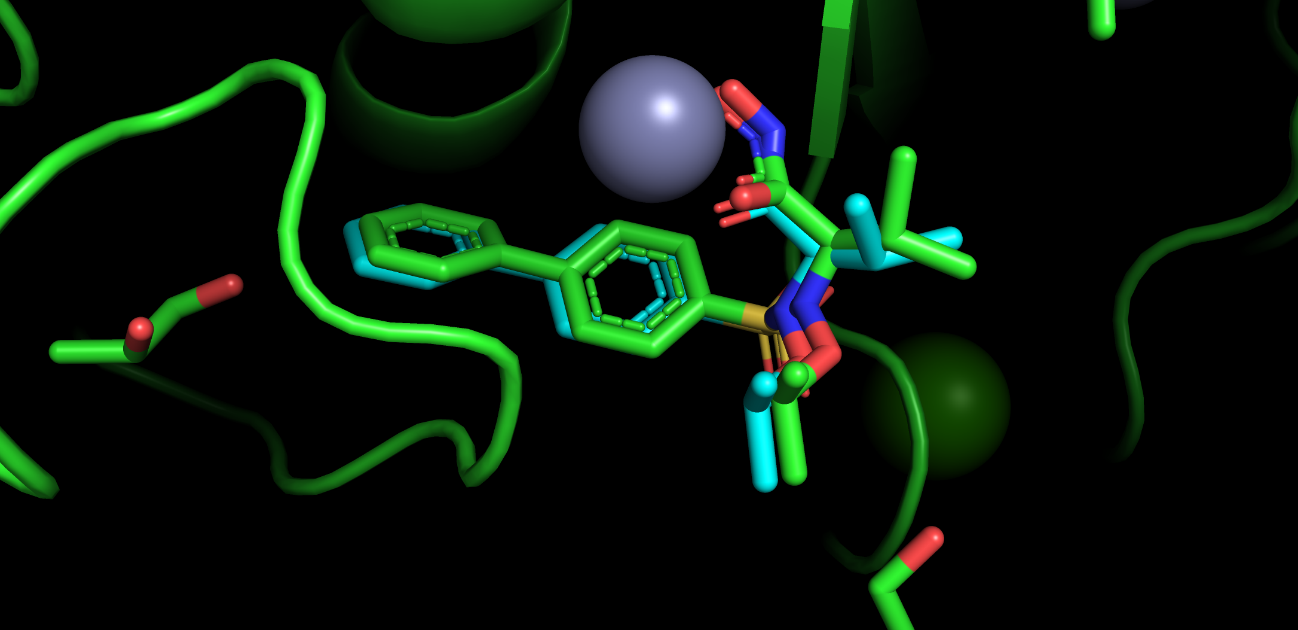


Figure S3. **Redocking validation of the crystallographic ligand in glycolate oxidase.**
Representation of the binding site of Glycolate oxidase showing the superposition between the crystallographic ligand and the docked pose of 4-carboxy-5-dodecylsulfanyl-1,2,3-triazole (CDST). The protein structure and the native co-crystallized ligand are shown in green, while the redocked ligand used for validation is displayed in blue. The redocking procedure yielded a binding affinity of −7.3 kcal/mol and a root mean square deviation (RMSD) of 0.269Å.


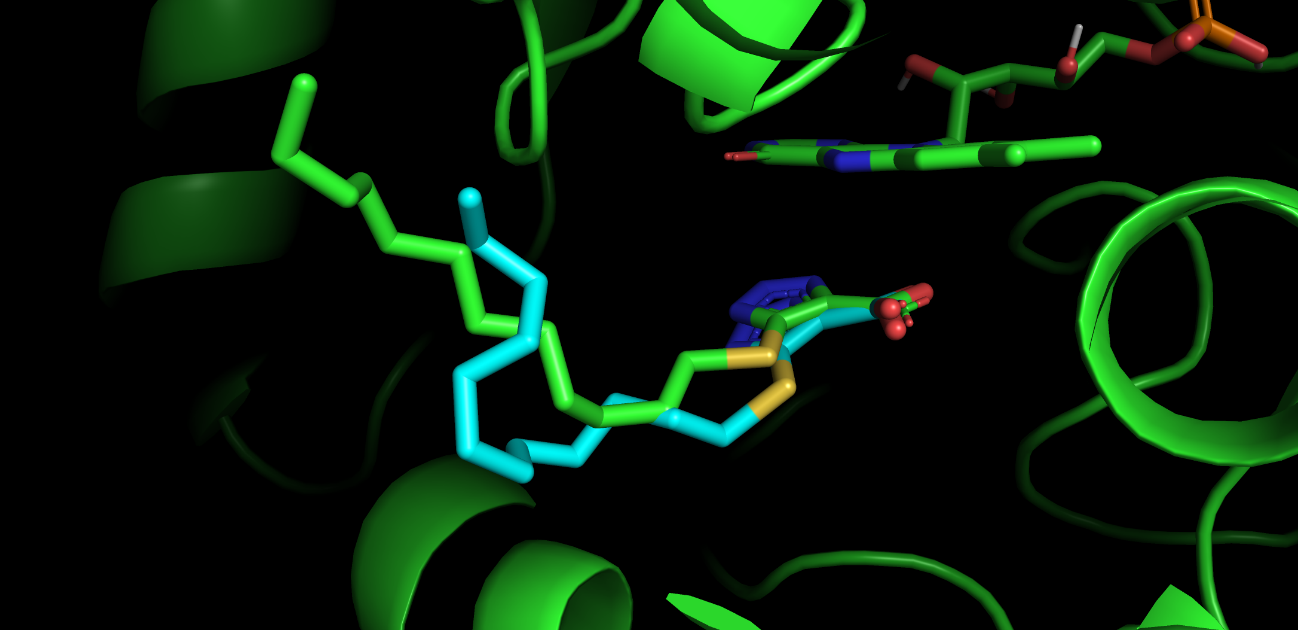


Figure S4. **Redocking validation of the crystallographic ligand in Phosphoethanolamine Cytidylyltransferase.**

Representation of the binding site of Phosphoethanolamine Cytidylyltransferase showing the superposition between the crystallographic ligand and the docked pose of cytidine-5'-monophosphate (CMP). The protein structure and the native co-crystallized ligand are shown in green, while the redocked ligand used for validation is displayed in blue. The redocking procedure yielded a binding affinity of −9.6 kcal/mol and a root mean square deviation (RMSD) of 0.446Å.


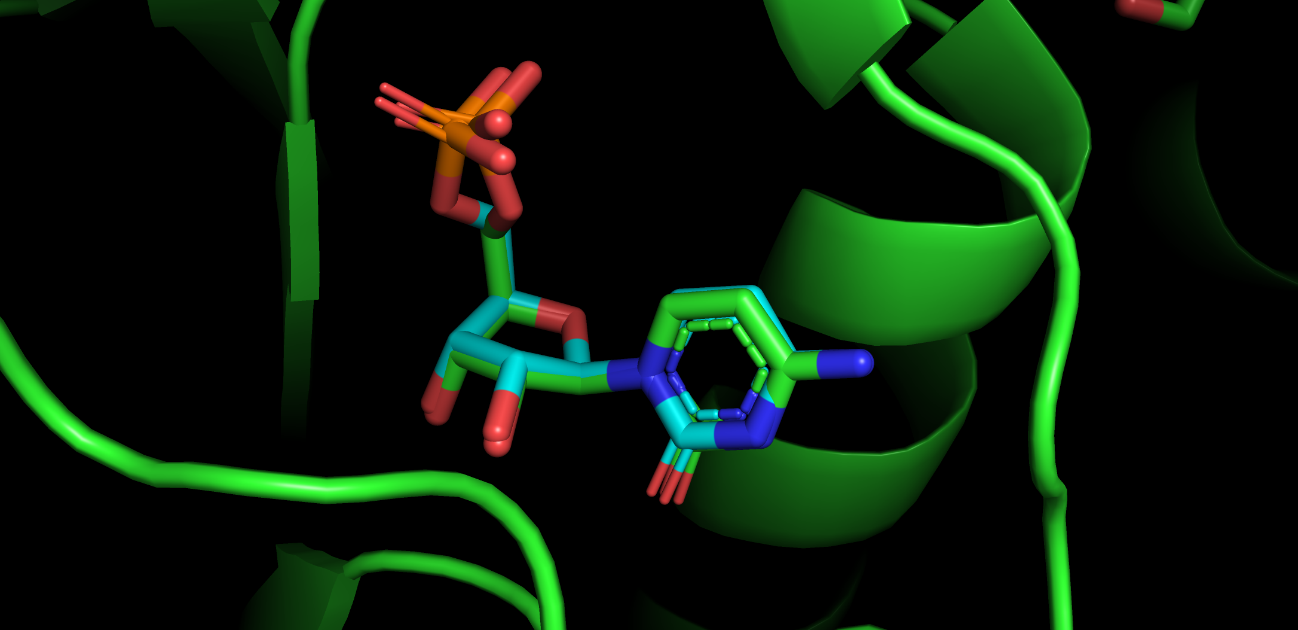


Figure S5. **Redocking validation of the crystallographic ligand in CaSR VFT domain.**
Representation of the binding site of CaSR VFT domain showing the superposition between the crystallographic ligand and the docked pose of cyclomethyltryptophan (TCR). The protein structure and the native co-crystallized ligand are shown in green, while the redocked ligand used for validation is displayed in blue. The redocking procedure yielded a binding affinity of −8.5 kcal/mol and a root mean square deviation (RMSD) of 0.581Å.


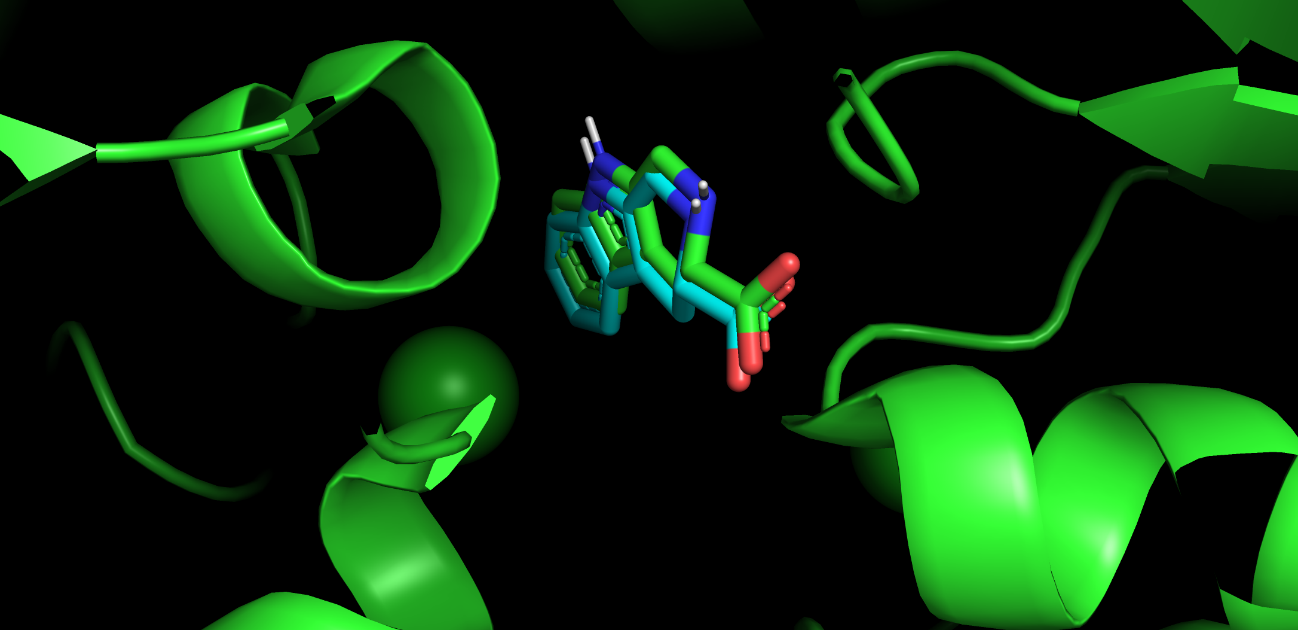


Figure S6. **Redocking validation of the crystallographic ligand in CaSR 7TM domain.**
Representation of the binding site of CaSR 7TM domain showing the superposition between the crystallographic ligand and the docked pose of 2-[4-[(3S)-3-[[(1R)-1-naphthalen-1-ylethyl]amino]pyrrolidin-1-yl]phenyl]ethanoic acid (Evocalcet). The protein structure and the native co-crystallized ligand are shown in green, while the redocked ligand used for validation is displayed in blue. The redocking procedure yielded a binding affinity of −9.5 kcal/mol and a root mean square deviation (RMSD) of 1.695Å.
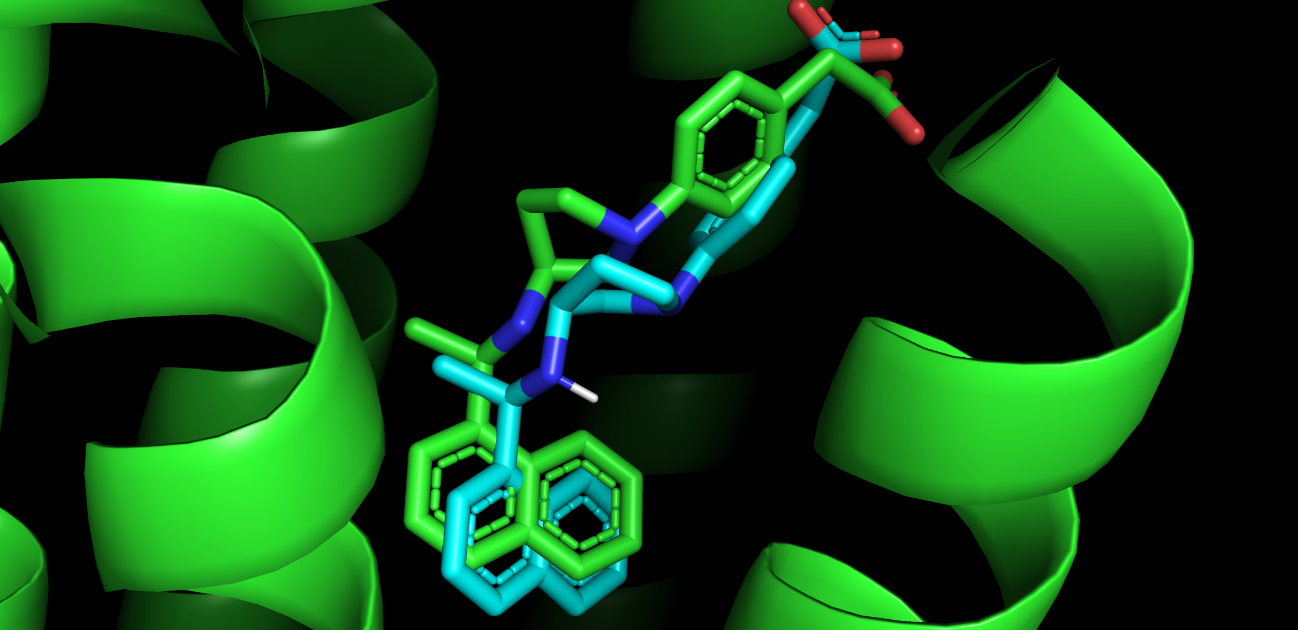

Supplement: Supplementary file 1 — Supporting File 1: cbdv71172‐sup‐0001‐SuppMat.docx [file CBDV-23-e03734-s001.docx]
